# Analyzing Yemen’s health system at the governorate level amid the ongoing conflict: a case of Al Hodeida governorate

**DOI:** 10.1007/s44250-023-00026-w

**Published:** 2023-05-10

**Authors:** Raof Al Waziza, Rashad Sheikh, Iman Ahmed, Ghassan Al-Masbhi, Fekri Dureab

**Affiliations:** 1 Institute for Research in International Assistance (IRIA), Akkon Hochschule für Humanwissenschaften, Berlin, Germany; 2 Yemen Public Health Network, Sana’a, Yemen; 3 World Health Organization, Gaziantep, Turkey; 4 Nanjing University of Science & Technology, Nanjing, China; 5 Heidelberg Institute of Global Health, Medical School, Ruprecht-Karls-University, Heidelberg, Germany

**Keywords:** Health system, Health system strengthening, Building blocks, Conflict, Qualitative study, Yemen

## Abstract

**Background:**

Yemen is regarded as one of the Middle East’s poorest countries. Decades of political, economic, and social difficulties have culminated in the current protracted conflict. As a result, the globe experienced its worst humanitarian catastrophe. The ongoing war has affected several public services, notably the health sector, which is operating at less than half its capacity. This study aims to examine Yemen’s health system at the governorate level (Al Hodeida) amidst the current conflict. It analyzes current challenges and produces suggestions for enhancement.

**Methods:**

The study used qualitative research methods such as Key Informant Interviews (KIIs) and document analysis. The study used WHO’s health systems framework to measure health system performance. Twelve KIIs were conducted via Skype with several health stakeholders. In addition, documents were analyzed to inform the subject guide, generate themes, and aid in the triangulation of results.

**Results:**

According to the study findings, the governorate health system managed to offer a minimum level of healthcare services while making some advances in outbreak control jointly with other partners. One of the main difficulties confronting the governorate’s health system is a severe lack of financial resources forcing it to rely entirely on external aid. Furthermore, other significant deficiencies include inadequate health system organogram, low reporting capacities, insufficient funding, and scarcity of health professionals.

**Conclusion:**

Yemen’s frail health system has been weakened by almost eight years of insecurity and conflict. If the current scenario continues, most of Yemen’s health system’s operations and indicators will likely deteriorate. On the other hand, progress in some areas, such as primary healthcare (PHC) services and disease management, is remarkable. However, for better performance, Yemen’s health system leadership and stakeholders should seek a holistic strategy to improve the entire dimensions of the health system.

## Introduction

The World Health Organization (WHO) defines the health system as all organizations, actions, and people whose core intention is to sustain, promote and restore health. In addition, it aims to improve the population’s health, enhance the health system’s responsiveness, and enforce financial fairness. Every country should regularly monitor these three essential goals to form the main base for the health system assessment facilitated by WHO [1]. Furthermore, health systems include the stakeholders, resources and institutions connected to the financing, regulation, and delivery of health activities. Moreover, this even leads to a broader definition of the health system, including efforts to improve road and vehicle safety, resulting in fewer road traffic accidents. Generally, health systems should aim to ensure proper provision of healthcare services and that they are accessible to everyone. Reaching this goal is a key to achieving the Sustainable Development Goals (SDGs) [2].

Attainment of SDGs for health has been a challenge for many low-income countries, particularly those in active conflict states such as Yemen. Yemen’s long history of political instability and enduring conflicts make it one of the poorest countries in the Middle East [3]. The ongoing conflict, with estimated 30.5 million Yemenis, has resulted in almost 20.7 million people in need of all aspects of humanitarian assistance. An Estimation of more than 80% of the Yemeni population lives below the poverty line representing the worst humanitarian crisis in the world [4]. Moreover, the conflict has led to a fractionated health system and incurred further collapse of the already fragile health system [5]. Prior to the current crisis, a total number of 5285 health facilities (HFs) were distributed throughout the country, with 90% of them fully functioning. Currently, only 54% of the HFs are fully functional, 40% are partially functional, and 6% are not functional. According to an assessment performed by a WHO initiative called HeRAMS (Health Resources and Services Availability Monitoring Systems), the reasons for the non-functionality are mainly related to inadequate financial resources, lack of health workforce, and shortage of essential equipment and furniture [6].

After more than six years of relentless conflict with intensified escalation since June 2018, Al Hodeida governorate was among the most affected ones. The impact of the crisis and natural hazards have undermined every segment of the governorate and led to an increased risk of famine, forced displacement, outbreaks and conflict causalities [4, 7, 8]. In addition, the disruption of electricity in almost all parts of the country because of the war did not only worsen the already-weak health system but also negatively impacted the functionality of hospitals and clinics, the cold chain for medicines and technologies as well as the lives and livelihoods of the population. Consequently, water supply, sanitation and agricultural services were all hindered, resulting in the deterioration of the entire nation’s health, nutrition and food security, especially for mothers and children [9].

This study aims to analyze the health system structure at the governorate level. Specifically, the study investigates views about challenges, gaps and opportunities in the health system in Al Hodeida governorate.

### Methodology

#### Study design

This study is a qualitative research based on key informant interviews (KIIs) conducted with experienced Yemeni health system individuals and documents review. A phenomenology approach was carried out for this study. There are several approaches to phenomenology; however, hermeneutic (interpretive) and descriptive phenomenology approaches are the two common approaches that direct the majority of psychological research [10]. The study systematically explored the true meanings of experiences and perceptions of the studied subjects to have an in-depth understanding of Yemen’s overall Health system situation. The research team used the WHO health systems building blocks framework [11]. This framework aims to create a common understanding of what health systems are and what accounts for health systems strengthening. Despite the limitations of the WHO building blocks, it is ubiquitously common and recognized by policymakers, programmers and global health researchers [12].

#### Study area

The study was conducted in Al Hodeida governorate, Yemen. Al Hodeida governorate is located in the western part of Yemen, a narrow coastal plain between the Red Sea and the foothills of the highlands. It is the country’s main port on the Red Sea, where commodities such as goods, food, fuel and humanitarian aid enter Yemen. It is considered the fourth largest city in Yemen in terms of population, which is about 2.7 million people, excluding IDPs [4]. It is a tropical region where the weather is characteristically hot and humid. Most people in Al Hodeida governorate live under the poverty line [13]. Despite the Stockholm agreement, which was purposed to restore peace, Al Hodeida governorate remains the most dangerous place to live. The ongoing conflict still hinders people from accessing several basic and life-saving services, including hospitals, water systems and food [14].

#### Data collection

Researchers organized data collection into two stages: the first was searching for and reviewing literature and documents, including grey literature, and the second was conducting key informants interviews. Those interviews included experts from the Governorate Health Office (GHO) and District Health Office (DHO) directors, health staff working in public health facilities and Non-Governmental Organizations (NGOs) staff. The research investigator collected and stored all relevant documents electronically in one folder and used a matrix to classify the documents based on their topic relevance to the study.

Documents analysis, including the review of available online documents and reports related to Yemen’s health system, were selected based on the following keywords*: “Yemen’s Health System”, “Health System Strengthening”, “WHO Building Blocks”, “Health Governance and Leadership”, “National health policy”, “Health Service Delivery”, “Health Systems in Fragile and Conflict States”, “Health Financing”, “Fragile Health Systems”, “Yemen District Health System”, “Ministry of Health, Yemen”, and “Health Information System”*. Moreover, electronic documents acquired from MOPHP and Al Hodeida GHO were reviewed as gray literature to reflect gaps and opportunities of the health system at a governorate level. Documents review and content analysis were helpful in elaborating the overall situation of the health system in Yemen. However, they did not provide enough information to answer the study questions and address the main objectives. Therefore, gaps produced from the deficiency of clear answers in the document analysis, such as health system structure at a governorate level, gaps and opportunities were filled from information abstracted from the interviews.

For primary data collection, twenty health informants were meticulously invited, depending on their background, to participate in the interviews via email. An interview guide was developed exclusively for this study to explore the study objectives (see Annex 1 for the interview guide). Twelve out of the contacted twenty individuals agreed to undergo and get involved in conducting the interviews, while the rest eight did not respond. A pseudonym for each participant (from 1 to 12) was given for anonymity purposes. Interview transcripts were analyzed using content analysis by reading each interview transcript and underlining striking statements, and then they were coded across each interview using the inductive analysis method. All codes were grouped into a few themes during the analysis process.

With this number of participants, the investigator noticed that no new information was coming up and, therefore, data saturation was reached. The below table (Table 1) shows the informants’ reaction to the invitation against their professional/ occupational background.

**Table 1:** No. of key informants participating in the interviews

Informants’ professional background	GHO	DHO	HFs	INGOs	Total
No. of invited informants	7	5	4	4	20
No. of informants who agreed	5	2	3	2	12
No. of informants who didn’t respond	2	3	1	2	8

The researchers employed purposive sampling to select key health informants to be interviewed based on their position within Al Hodeida governorate health office, HFs and NGOs. Initially, participants were approached and given the information sheet and consent form of the study prior to the interviews. After obtaining key informants’ consent to participate in this study, the interviews were conducted using an interview guide developed explicitly for this purpose. Following COVID-19 public health protection measures, interviews were conducted online using Skype. Conducting such an approach created a limitation for the principal investigator to take notes on body language. However, this challenge was reduced by paying extra attention to intonation throughout the interviews. The interviews were carried out in Arabic language and then translated into English. Hence, the data was rendered in English for further analysis. Participants’ anonymity and confidentiality were ensured using coding numbers for data analysis.

##### Limitations

This study focused on analyzing the Yemeni health system at a governorate level, considering the public sector; therefore, the private sector was excluded.

## Results

This study focuses on the current health system status at a governorate level. It aims to develop a general understanding of the health system in Al Hodeida governorate. From the literature review of existing relevant documents and the interviews with key informants, the results of this study emphasized mainly on challenges and gaps of Al Hodeida health system. The analysis applied the six building blocks of the WHO health systems framework: leadership and governance, health information system, health financing, health workforce, service delivery and access to essential medicine.

### Health system building blocks

#### Leadership and governance

The health system in Yemen is organized administratively at three levels, namely central, governorate and district. This system was characterized by a centralized structure and management until the early 2000s when the ministry endorsed decentralization policy as part of the health sector reform strategy and the local authority law by the government then. While the central ministry is responsible for the overarching policy and strategy development as the top level, the governorate health office at the middle level and the district health office at the distal level are responsible for the organization of healthcare service delivery functions such as planning, budgeting and human resources management among others. In Al Hodeida, the governorate health office (GHO) supervises and manages 26 district health offices [one district health office (DHO) for each district].

**Fig. 1:**
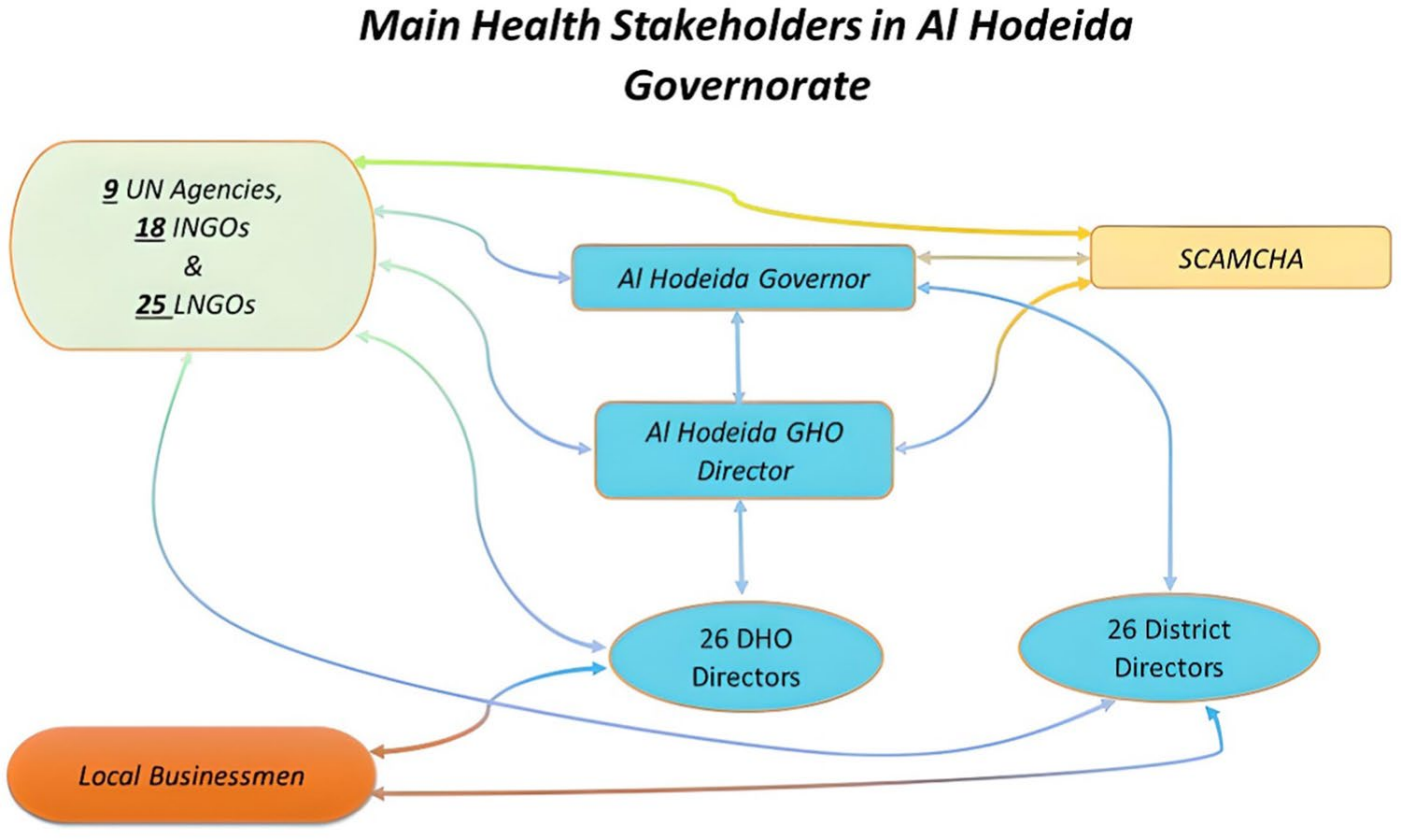
Key stakeholders et al. Hodeida governorate

In Al Hodeida governorate, a diverse number of players are involved in the health sector. All informants agreed unequivocally that the health system's decision-making is decentralized. The governorate’s health officials and local administrative authorities make most decisions. The DHO director, together with the district director, has specific responsibilities, such as monitoring the HFs, allocating medicine amounts, and overseeing community engagement. What is beyond their control, however, is escalated to the governorate level for discussion and guidance. To implement programs and initiatives, all NGOs must first engage with the SCAMCHA. Then, project regulation must be coordinated with Al Hodeida GHOs and DHOs, with regular communication between NGOs and SCAMCHA for permits and approval for field visits and training, see Fig. 1.

An informant described the decision-making by stating:

*“Decision-making is influenced by the decision itself and its context. It may be described as both centralized and decentralized. MOPHP instructs decisions that must be implemented for the entire country to GHOs in all governorates for implementation. COVID-19 decisions, for example, are made at the central level. GHOs are kept regularly updated by MoPHP”*** Informant** # 1- NGO

Another informant elaborated on the decision-making at the governorate level by saying:

*“The GHO manager makes governorate-related decisions, sometimes in collaboration with the governor and local authorities. Decisions surpassing the GHO’s manager boundaries are advanced to MoPHP”*** Informant** # 11- GHO

According to the majority of informants, Al Hodeida GHO lacks a clear organizational organogram reflecting levels of supervision and is afflicted by several difficulties that limit its ability to carry out its purpose effectively. One informant stated:

*“Al Hodeida GHO overlooks many things. For example, there is no organizational chart outlining the GHO system and its functions when it comes to management”*** Informant** # 5-GHO

Since the political situation deteriorated in 2011, the structure of Al Hodeida GHO has become inoperable. Due to factors such as inadequate financial resources and a lack of competent personnel with adequate capacities, this essential sector was undermined. Furthermore, NGOs grabbed this department’s function in collaboration with a newly created governmental organization, namely the Supreme Council for the Administration and Coordination of Humanitarian Assistance (SCAMCHA). An informant from GHO has stated that:

“The situation is no longer the same as it formerly was. The department of emergencies and operations center (EOC) is not functioning. Since the establishment of SCAMCHA, all NGOs have to go through this new institution to oversee their plans and emergency operations”** Informant **# 11-GHO

National health strategies are overlooked and are not adequately understood, if at all. Only two of the eight interviewees were aware of some strategies, while the rest were either unaware of them or referring to unrelated topics. An interviewee from GHO has indicated an example of a health strategy by saying:

*“There are reproductive health strategies supported by UN agencies and a local non-profit institution to reduce mother and neonatal morbidity and mortality.”*** Informant** # 8-GHO

Several issues, including a lack of financial resources, competent human resources, the influence of international NGOs, fragmentation of the health system and managerial shortcomings, have weakened the leadership of Al Hodeida GHO. The COVID-19 outbreak in Yemen demonstrated, along with other factors, a low level of openness and transparency by MoPHP. The MoPHP in the North opted to pursue the step of issuing an official denial of COVID-19 exact positive cases leading to diminishing people’s trust and faith in health authorities and increased infodemic and rumors’ spread [15]. When questioned about communication and coordination between the two conflicting parties, most key informants responded that none exists. In contrast, only one informant stated that communication with all districts is still ongoing. One of the participants explained that clusters are the leading coordination platform at the governorate level.

*“Clusters are usually performed monthly. UN agencies are taking the lead, inviting people from GHO as main partners, of course, but the conduction and timing of the meetings are on the power of those UN NGOs because of their huge funds, as you know”*** Informant** # 2-NGO

Other interviewees indicated communication and reporting from districts within the jurisdiction of Al Hodeida governorate:

*“There are some parts within the district controlled by AlSharaia [legitimate authority], but there is not any kind of communication.”*** Informant** # 4-DHO

*“All 26 districts are reporting to the GHO on a monthly basis, and communication takes place through our DHO directors”*** Informant** # 8-GHO

Considering the vast funds from different donors and the presence of many stakeholders, an opportunity should be shifted towards enhancing the capacities of personnel in the governorate health office. Moreover, a better advantage of cluster meetings has to be used to empower the leadership et al. Hodeida governorate to update and implement national health policies, strategies and plans.

#### Health information system

**Fig. 2:**
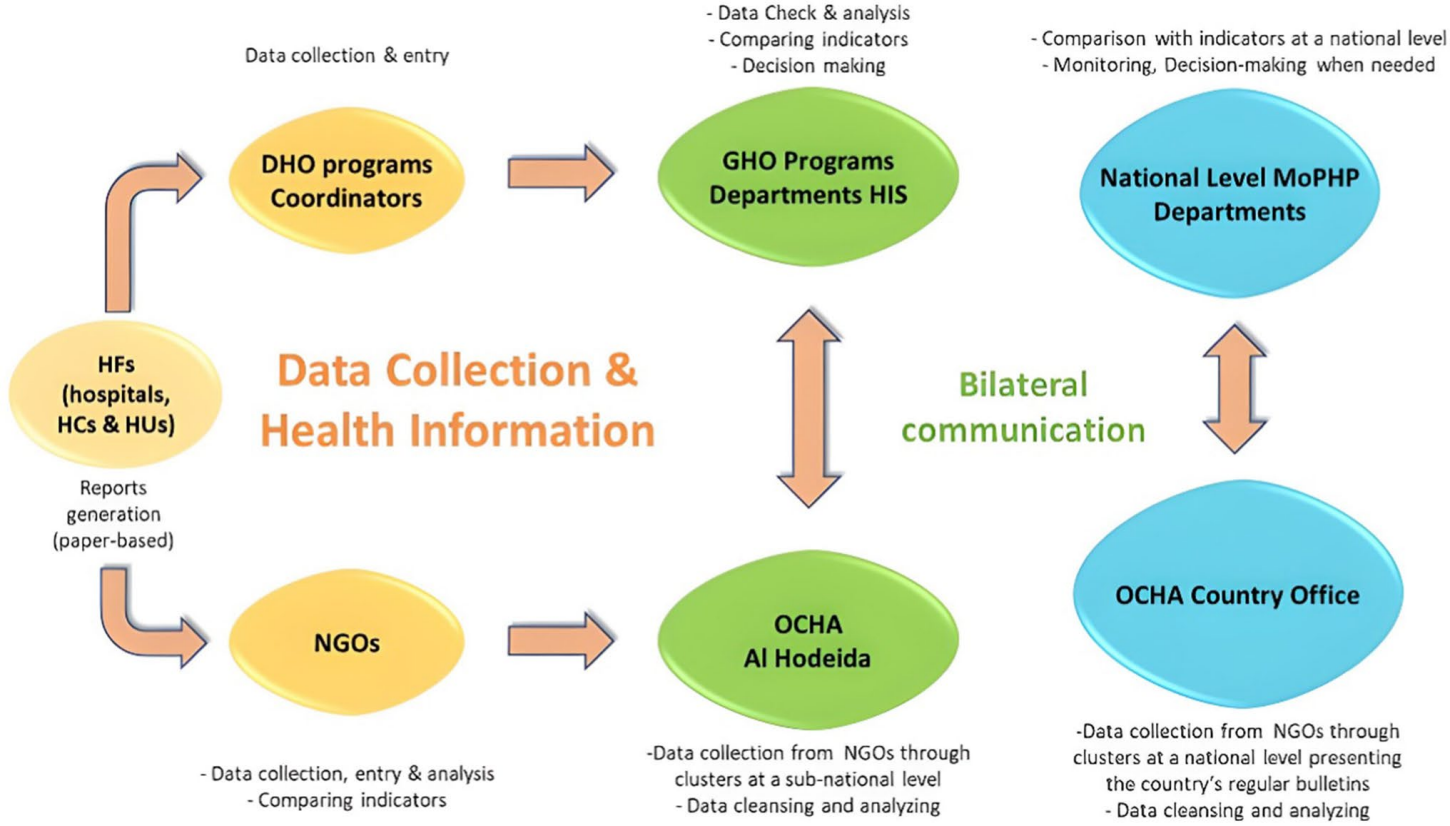
Health data flow at a governorate level

The reporting system has two parallel routes. The first is the governmental route, in which reports are gathered monthly using the standard registers and reporting forms for each health program. The second path is the NGOs’ track, in which the HFs are required to submit several report forms in order to meet the needs of the sponsoring NGOs. Figure 2 was derived from qualitative interviews and the investigators’ reflections on their hands-on experience. It clarifies the reporting system’s flow from HFs to the county level. An informant gave an example of data flow across different levels:

*“At the district level, every healthcare facility has different registries in which they manually report communicable diseases data, as an example, for the eDEWS surveillance system on a monthly or weekly basis. The program coordinators assemble these reports and present them to the DHO. Next, the DHO submits these findings to the GHO, who then forwards the assembled reports to the MoPHP at the central level”*** Informant** # 4-DHO

Another interviewee from a health facility indicated the double efforts of reporting to the government and NGOs through different channels by saying:

*“We are required to submit two reports: one for the district health office and one for the NGO that is supporting the health center. The NGO occasionally provides us with forms to fill out that are not the same as the DHO’s”*** Informant** # 3- HF

Al Hodeida’s GHO’s health information system lacks the essential equipment and personnel for adequate functionality. According to the majority of informants, several issues, including infrastructural restrictions, capacity-building deficits, and a reliable reporting mechanism, are responsible for the health information system’s fragility. One of the interviewees indicated to the weaknesses aspect of information systems:

*“There is a health information and statistics department, but it lacks basic equipment such as computers and an internet network. Until now, reports are completed manually in HFs, and data entry is performed and sent to the GHO through flash disks through DHOs departments’ coordinators.”*** Informant** # 11-GHO

Another informant elaborated on an example of challenges in reporting deaths at a district level:

*“In general, the reporting system at a district level is good, but something is missing. The number of deaths and causes of death is not well documented.”*** Informant** # 4-DHO

While the implementation of the eDEWS system added a great value to the health information system detecting the early spread of communicable diseases, an opportunity is forecasted to take place if similar systems can be introduced collaboratively with UN agencies for the surveillance of non-communicable diseases.

#### Health financing

Before the complicated situation in Yemen, annual total health expenditure (THE) was among the lowest in the world. Health spending per capita grew from $25 in 2000 to $80 in 2014, then fell to 72$ in 2015. According to most sources, the government’s financial support for health is now severely constrained by the long-lasting war. In general, private health spending, including out-of-pocket (OOP) payments, has grown substantially, accounting for 43.5 percent of expenditure in 2000 and reaching 81 percent in 2015. Donor assistance for the health sector is one of the most critical factors for the preservation of health services in Al Hodeida governorate. The provision of operational funds, incentives and training for health workers, and medical supplies ensure the availability of health services. A key informant described the financial situation challenges at a rural health hospital by saying:

*“The GHO funding for a rural hospital is quite small and insufficient. For example, in the budget, 60,000 Yemeni Riyals (YRs) are allocated aside for gasoline each month. A barrel of fuel costs around 70,000 YRs, and the daily usage in Al Hodeida, owing to the hot weather, is two barrels. As a result, the disparity is enormous.”*** Informant** # 4- DHO

A sharp decrease in humanitarian aid is noticeable from 2020 onward, where about 82% of the needs required are unmet [16]. This will subject many lives to be at risk. According to one informant, a shortage of appropriate humanitarian aid will aggravate the already defined worst humanitarian crisis in the world.

*“This year, the fund we have is almost 40% of what we had the previous year. Not only were employees sent home, but we had to adapt our plans prioritizing life-saving activities and stop some of our projects in some areas.”*** Informant** # 2-NGO

#### Health workforce

Al Hodeida GHO faces an immense challenge concerning the health workforce and retaining them, especially after the escalation of the conflict. Currently, general practitioners and specialists, in particular, represent only 5 percent of the total health staff in the governorate. The gaps in the number of health workforce are enormous even prior to the conflict. In 2014, data showed that the gaps in physicians were 47 percent, more than 80 percent for nurses and midwives, 96 percent for dentists, and 78 percent for pharmacists [17, 18]. From a gender perspective, nurses and midwives show an equitable distribution, while physicians are dominantly males. Five out of twelve informants stated that many doctors migrated to other areas within the country or outside Yemen due to the intensified conflict in the governorate. This has led to a decrease in the provision of healthcare services, especially for those in need of special treatment.

*“Before 2015, many health workers (HWs) and physicians who worked in private hospitals and performed surgical procedures and specialized health services were from other cities in Yemen. When the conflict erupted, the bulk of people fled to areas outside of Al Hodeida, leaving a significant gap in the governorate”*** Informant** # 5-GHO

Health institutions in Al Hodeida continue to provide education to students (six main institutions). Annually the average number of graduates is 1355. Out of this number, 130 medical doctors, 265 nurses, 310 laboratory staff, 215 midwifery personnel, 125 dentists and 310 pharmacists. This is foreseen as an opportunity to back up the health system in Al Hodeida in compensating the leaked health personnel and somehow support in covering the gap which the governorate suffers from currently.

The major impediment is the conflict, which reduces access to health care, disrupts the health payroll system and increases employee turnover, which multiplies the load on the remaining personnel. In addition, the health cadre’s capacity to deliver quality health services is hampered by inadequate working conditions and the volatility of incentives.

*“In the Health Unit (HU), we confront several problems. For instance, the number of persons visiting the HU is large, and we only have two rooms for treatment, nutrition, reproductive health, vaccination, and a corner for storing medications and dispensing [...] government wages are now paid every three months”*** Informant** # 10-HU

#### Service delivery

Healthcare services delivery is organized at four levels as described by Yemen’s national health strategy, namely health units and health centers as the first level, governorate and district hospitals as the second level, a small number of referral hospitals as the third level and fewer specialized centers and hospitals as the fourth level [19]. Annex 2 elaborates more on the distinction between levels of health care and the services provided.

The governorate of Al Hodeida has 423 public health facilities accredited by the Ministry of Public Health and Population (MOPHP). Health units and health centers represent the majority, with a bulk number of 401. They offer primary, preventive, and limited secondary curative treatment, as well as health promotion services. A secondary level of three inter-district hospitals (in the districts of Aldhahi, Hais, and Bayt Alfaqih) and 14 district hospitals providing a more extensive range of secondary and tertiary services. Al-Thawra Hospital, classified as a general authority hospital at the governorate level, acts as a referral point for specialist health services as well as a teaching facility for medical colleges. Furthermore, specialized centers, such as dialysis centers, a mental health facility, a cancer treatment center, a blood bank and a laboratory reference center, are primarily concentrated in the city’s center of Al Hodeida governorate. Table 2 presents the type and size of health facilities in Al Hodeida governorate.

**Table 2:** Type and number of health facilities in Al Hodeida governorate

Type of health facility	Number	Percentage (%)
Health units	339	80
Health centers	62	14
District/Rural hospitals	10	2
Inter-district hospitals	2	0.5
Maternal and child centers	7	1.5
Other specialized centers	3	0.7

Good healthcare service delivery is ideally the one that is safe, effective and of good quality. It should be delivered to all people in need at a low cost. Quality of health care services has several definitions; however, WHO has defined it as the degree to which health care services for populations increase the likelihood of desired health outcomes [20, 21]. According to most respondents, the quality of care in public institutions is unsatisfactory, but the quality of healthcare services in private facilities ranges from poor to relatively good. Due to limitations and unfairness in obtaining specialist medical care, people are forced to either bear the financial burden of seeking treatment in distant locations or look for an opportunity to acquire it within the few options available. The deepening of the conflict, along with the exodus of professionals from Al Hodeida governorate, resulted in the preservation of just basic health services. Reproductive health, vaccination, nutrition, integrated management of childhood illness (IMCI), and minor surgical operations are still provided in nearly all districts. However, secondary and tertiary healthcare services are in short supply [6].

*“Currently, there is only one public hospital in the governorate that provides good health services, which is Al Thawrah Hospital. As for the other public rural hospitals and both secondary and primary healthcare levels, they only provide essential services which are necessary for the current emergency situation. There are a minimal number of private hospitals in Al Hodeida, which sometimes people who can afford it prefer to go to”* **Informant** # 1-NGO

In response to the partially collapsed health system, the MoPHP adopted the minimum health service package (MSP) project in conjunction with WHO in 2017. This approach was devised to make healthcare delivery more accessible to most people. It consists of eight components aiming to improve the delivery of primary and secondary healthcare services. This strategy has been used in Al Hodeida, although only in a few areas. An opportunity can be created to include all other districts of Al Hodeida governorate with this approach to provide enhanced access to health services to all residents.

*“Health services, in general, are getting better recently, especially after the Ministry of Health came up with the MSP guideline. For instance, the reproductive health situation in Al Hodeida is very good now. Health workers have been trained very well on almost everything, especially handling emergencies”* **Informant** # 12-GHO

In 2019, Al Hodeida GHO conducted an assessment to identify the gaps and requirements of the governorate’s health facilities. The assessment included 339 Health Units, 62 Health Centers, and 12 hospitals. More than half of the Health Facilities, however, required rehabilitation as well as furnishings and equipment. Regarding the provision of healthcare services, only 32 percent of healthcare services are fully available, 40 percent are partially available, and 28 percent are not available [6]. The conflict has amplified the decline of healthcare delivery. Most respondents indicated that access to healthcare is determined by geography and the services sought. Conflict-affected zones and rural areas are disadvantaged in Al Hodeida governorate. The escalation of the political situation and the influence of the de-facto authorities on MoPHP policies in the North influenced some programs of healthcare provision. For instance, family planning services deteriorated and have been overlooked since the upsurge of the crisis in the governorate. A key informant indicated difficulties in accessing services:

*“Considering the country’s current situation, primary healthcare services are doing fine. People living around the first level get to health facilities easily. However, those living in the second and third levels do not make it easily due to the distance between the villages and Health Units”* **Informant** # 10-Health Unit

Another informant from a health center indicated the challenges in providing reproductive health services in Al Hodeida:

*“Family planning used to be better before because we provided consultations and provided the most appropriate contraceptive to each woman. This is bad now because family planning services and supplies have been scarce. People purchase contraceptives from private pharmacies and use them randomly without professional consultation”* **Informant** # 3-Health Center

#### Access to essential medicine

Prior to the conflict, governmental health financing and accessibility to vital medicines were critical concerns in Yemen. It has worsened as the economic situation continues to deteriorate and the ongoing conflict enters its eighth year. Before the 2011 Uprising, there was a governmental expenditure on essential medicines, according to three informants. The downfall of the Supreme Board of Drugs and Medical Appliances (SBDMA) at a central level within MoPHP, both financially and administratively, has led to a complete failure countrywide to provide the essential drugs to the health facilities.

*“Currently, there is no financial provision from the government for purchasing medicines and supplies. Previously, the national drug supply program used to purchase essential medicines through a public government tender once a year and was financed by the government. Donor countries were supporting this program with large sums, but it stopped more than ten years ago”* **Informant** # 8- GHO

Supply chain management and access to essential medicines have been a vexing issue both at central and governorate levels. According to some key informants, a persistent lack of regulation and coordination of medical supply between donors and GHO authorities lead to duplications and inadequacy in meeting the actual needs. Moreover, poor governmental investment in infrastructure results in improper medical storage, hindering people’s accessibility to safe and effective medicines.

*“There are zero communications or coordination to provide medicines according to the needs. For example, some NGOs implement projects directly at a district level and provide medicines to a district without checking with GHO about what to provide or what is missing. Therefore, there is usually an issue where GHO supports with Amoxicillin, for example, for the district and the NGO provides the same”* **Informant** # 8-GHO

*“We always suffer from issues such as delays because of transportation and the long process it takes. Also, warehousing where in Summer electricity gets as high as 43-45 degrees. Then only we can run the generators, for example, three hours in the morning and the same in the afternoon”* **Informant** # 7-DHO

## Discussion

The purpose of this research is to get a broad understanding of the health system in Al Hodeida governorate, as well as to identify and analyze system issues and gaps using the health system building blocks framework. Analysis of collected data shows serious challenges and gaps in the health system at the governorate level across all building blocks. The study reinforces previous research findings on the harmful impact of the ongoing war and instability on Yemen’s health system structure [22]. However, this study documents health system issues at the decentralized level.

The presence of two administrations within the same governorate has a major influence on the already vulnerable health system, and as a result, inefficiencies are unavoidable. The instance of Iraq, where the establishment of Iraqi Kurdistan as a de-facto state led in the fragmentation of the health system [11]. Variations and gaps in the health system were caused by changes in leadership and power between the two parties. For example, having deficiencies in the health information system because of fragmentation impedes important tasks like policy formulation, evidence-based decision-making, monitoring and assessment of the public health situation, and sensible resource allocation [12]. Although the health system in Yemen has invested in establishing a district health system during the 2000s as part of the health sector reform strategy [23], there has been no reflection from any informant or recent literature on using this as a framework for supporting or operationalizing health system at the distal level.

MoPHP is committed to showing the public and the government how well it operates towards securing the continuance of healthcare delivery reinforced by openness and accountability [19]. The concept of accountability, being held accountable to act strictly to standard ways of behavior and obligated to justify failures, is utterly absent in the Arab world [24]. Even before the beginning of the conflict, a study of health governance in Yemen revealed a few accountability issues among other governance dimensions [25]. Another additional burden is the fragmented health information system which has been for long characterized by inefficiency, inaccuracy and low quality coupled with weak monitoring and evaluation practices [22, 26–29]. Dual reporting of health data to government and NGOs using parallel channels is widening the health system fragmentation and overburdening the health workers.

From an ethical perspective, it is unclear how decision-making, scarce health resources control and allocation are managed at the health system’s different levels, particularly with low transparency and accountability. Additionally, results show that strategic plans and policies are likely vague to the majority of health staff at different levels of the health system. Probably such a finding is due to MoPHP’s weak communication, absence of clear policies and strategies or low capacity of health staff to comprehend and operationalize them at decentralized levels. Moreover, the presence of several national and international players to respond to emergency requirements creates competing agendas for the health system at the governorate level. Inadequate coordination and communication result in an unbalanced response and misdistribution of already inadequate medical and equipment supplies.

Changes in political power had a devastating impact on all governmental institutions, including the health sector. Recurrent turnovers of qualified personnel and replacement with others who are insufficiently qualified stymie the healthcare system’s growth. Similarly, in Timor-Leste, multiple political transitions resulted in personnel turnover at the Ministry of Health. Those new ones lack adequate technical and management competence, limiting their ability to provide basic and essential services [30].

The dependence of the current health system on donors’ funds resulted in marginalizing the National Emergency Operational Center’s (EOC) role. The stream of funds pledged to respond to the current worst humanitarian crisis created an impulse to control it. Therefore, the new institution SCMCHA was established nationally with a branch in every governorate in Northern Yemen [31, 32]. Thus, in Al Hodeida governorate, almost all emergency response and humanitarian aid are regulated through this new institution. This, in part, has diminished Al Hodeida’s EOC department functionality in the governorate health office and its mandate to plan, prepare and respond to health emergencies. On the other part, SCMCHA could play a significant role in emergency multisector coordination and harmonization, which is highly needed during conflict times. However, the argument is how this objective can be ensured neutrally while capacity and transparency in this institution are questionable.

Financing public health services come from three sources: donors, the government’s allocation for health and privately through beneficiaries’ contributions. While government financing of health is irregular and solely for wages, the donors’ funds remain the main source. Public health facilities apply user fees for health services mainly due to insufficient funds, and with diminishing humanitarian aid, people might be under additional burden, especially the poor. Ambiguity is high in the volume and use of these OOP payments in public facilities. Probably curative services such as those of non-communicable diseases (NCDs) are the main service that patients must pay for when seeking public health services. NCDs’ support in the humanitarian context might be overlooked in Yemen, and more focus is given to primary and preventive healthcare services. Alternative health financing methods, such as a well-designed health insurance mechanism tailored for the Yemeni context, should take the optimal attention from all actors as an exit strategy for donor-dependent funding and possibly toward peacebuilding efforts.

Throughout the years, MoPHP has adopted several strategies to improve and secure the provision of therapeutic, preventive, and rehabilitative healthcare services. A substantial diminution is readiness and preparedness to respond to emergency health needs such as epidemics, prevention of injuries and disabilities, and emergency services. For instance, the national health strategy has indicated the need for an emergency strategic plan and disaster medical stock that covers a period of not less than three months [19]. Unfortunately, this concept and its applicability on the ground are missing. However, COVID-19 seemed to have brought about opportunities. The already developed surveillance system and lessons learned from previous epidemics e.g. cholera and diphtheria outbreaks, helped a lot in minimizing the effect of COVID-19 spread. In coordination with UN and international NGOs, around 333 rapid response teams were trained to undertake cases’ testing and contacts tracking. In Al Hodeida governorate, two isolation centers and an ICU in the central districts of Al Hodeida governorate (AlHawk and AlHali) were equipped to respond to any suspected cases within the governorate. This, however, was insufficient and inaccessible for patients from the peripheral and conflict districts [7, 33, 34].

The Yemeni health system is structured widely by a vertical program approach focusing on the prevention and control of diseases countrywide, supported technically and financially by international NGOs such as WHO, The World Bank, and GAVI [35]. Despite the continuation of the conflict and the fragmentation of the health system, primary healthcare services are still provided in many health facilities, excluding conflict zones [36]. Available health workers continued to provide services in public health facilities. Fear of losing governmental posts, formal and informal user fees could be the motivating factors. Additionally, it has become a social norm in Yemen to give a small amount of money to healthcare providers in favor of getting extra care and attention.

NGOs play a crucial role in supporting humanitarian and emergency relief activities in conflict-affected areas. For example, NGOs have successfully delivered services and pushed for international commitments to aid Iraq throughout the war that began in 2003 [37]. Likewise, a study in Africa has revealed good outcomes of NGOs’ role in addressing risks impacting residents’ health, such as WASH and health services [38]. However, in a fragile context, NGOs can negatively influence the health system, such as in Somalia, with diminishing government oversight on health services and dependency on external aid [39]. Similarly, at the governorate level, health operations have become dependent on external funds and NGOs’ support.

Supplies and management of essential medicines continue to be a major challenge for the health system in Yemen [4, 22, 40–43]. Irrational use and prescription of medicines, including antibiotics, are widely common and evident [15, 44–46]. Challenges related to warehousing management and logistics of medicines and health supplies are enormous, mainly due to limited capacities in both human resources and infrastructures and weak coordination and harmonization between different health partners. Investment in strengthening the supply chain management seems inadequate and not prioritized by actors at the governorate level and most probably by central supporters too. The supplication of supplies and stockouts reported in this study indicate severe challenges in managing health commodities in the health sector. Evidence was found indicating that poor planning and coordination result in products being either out of stock or overstocked [47]. Such findings from the governorate level might increase the risk of losing supplies and hence diminish health system performance and efficiency. Information and data on health medicines and supplies management are trivial and difficult to access. A factor here is the underdeveloped supplies management information system that can capture a comprehensive picture of all supplies at governorate, district, and facility levels.

## Conclusion

Active war and instability have affected the health system negatively at a decentralized level across all its building blocks. With weak coordination, inadequate communication and competing agendas at the governorate level, missed opportunities exist, leading to unavoidable inefficiencies. Integration of health information systems is a priority to avoid overburdening health workers and further fragmentation of the health system. Primary health services are donor-dependent financially and are maintained at a minimal level, yet specialized services, including those for NCDs, are widely not accessible or absent in public sector facilities. The scarcity of a well-qualified workforce has subjected the health system to shortfalls and defects. User fees are applied in public facilities and need careful examination of their effect on accessibility, especially for the poor.

## Recommendations

While the study highlighted several gaps and challenges in the current health system, it also underscored several opportunities to strengthen its response during humanitarian time. Three-pronged recommendations are highlighted for health system improvements in Al Hodeida governorate. First, empowering the independence of the health system and response through a comprehensive focus on the overall national health policies and strategies and not only on emergencies-related ones. Second, strengthening the local health system by prioritizing and providing essential capacity building and infrastructures, including health information system equipment. Third, there is an urgent need to stop the conflict and restore peace to improve system performance.

## Data Availability

The data was obtained from several published documents, reviews and reports related to Yemen’s health system. To fill the gaps and meet the study’s objectives, further data were abstracted and analyzed through the conduction of key informant interviews.

## Annex 1

Interview Guideline.

Target people: staff of governorate and districts health offices, staff of health facilities & Staff of NGOs.

**Table:**

Building blocks	Questions (Health system structure)
Governance and leadership	1- How are decisions taken in general at governorate levels GHO, DHO, HF? Prop: Is the decision independent based on the situation of the governorate? Is there any influence from central level or other NGOs? Give example for a decision taken due to a health event 2- What is the influence of the health system fragmentation within the same governorate? Prop: Is there any coordination between all districts? example 3- Can you describe GHO’s organogram et al. Hodeida governorate? Prop: How is the coordination between the different departments done both functionally and administratively? *** Gap, Challenges, Opportunities
Health financing	5- What financial resources does the health system have to keep it running? Prop: (Government budget, Support from Donors, out of pocket) 6- What are the changes related to health sector financing? What are the opportunities? *** Gap, Challenges, Opportunities
Workforce	7-How did the on-going conflict affect the health workforce (Health cadre)? Prop: Turnover, Lay health workers 8- How often are HWs trained (annually) on the health services they provide? *** Gap, Challenges, Opportunities
Essential medicine	9- Is the vaccination and essential medicine available at health facilities? Mention them… Prop: Is it distributed free of charge ex. Insulin and other anti-diabetic drugs 10- How is supply chain management for health supplies managed? Prop: Is there proper supply chain process in MoPHP, is there a national guideline used? How are the supplies stored or transferred? Give example from EPI *** Gap, Challenges, Opportunities
Health services	11- What is the influence of the war on health care delivery? Prop: What is the percentage of health facilities which are working well? What kind of health services available and what is not ? 12- Regarding people’s access to health care services, what areas within the governorate are hard to be reached? 13- In your opinion, what are the challenges that prevent beneficiaries from reaching to health care services? *** Gap, Challenges, Opportunities
Information	14- How is the health information system working at the governorate level? Prop: Is the information accurate and reliable? Mention the tools to gather the information, mechanism of performing and reporting, give example of good reporting process performing *** Gap, Challenges, Opportunities

## Annex 2

Levels of health care in Yemen.

**Table:**

Level of Care	Facility/Service provision	Services
Community	Home (CMF, CHV)	Home visits, IEC, home deliveries
	Community facility (CHW, CHV)	Limited curative care, IEC, SAM screening and management. FP (condoms)
	Mobile team	Limited curative care including IMCI, ANC/PNC, EPI, SAM screening and management, FP (short-acting methods), IEC
Primary health care	Health unit	Limited curative care including IMCI, ANC/PNC, EPI, SAM screening and management, FP (short-acting methods), IEC. Refill of NCD prescriptions
	PHC centre	Curative care (OPD) including IMCI, TB and others, ANC/PNC, FP, EPI, SAM screening and management, NCD management. Normal Deliveries (selected facilities). Essential Newborn Care. Basic Laboratory
Hospital	District hospital	Curative care including OPD and Inpatient. Round-the-clock ER. IMCI, TB, NCD. ANC/PNC, Normal and complicated deliveries. BEmONC (selected facilities). EPI. SAM with medical complications. Basic laboratory. Essential Newborn Care + management of sick and LBW newborns (selected facilities)
	Inter-district hospital and Governorate hospital	Curative care including OPD and Inpatient. Round-the-clock ER with trauma care. IMCI, TB, NCD. ANC/PNC, Normal and complicated deliveries. BEmONC and CEmONC. Essential Newborn Care + management of sick and LBW newborns. EPI. SAM with medical complications. Laboratory and Radiology
